# Sorafenib maintenance after hematopoietic stem cell transplantation improves outcome of FLT3–ITD-mutated acute myeloid leukemia

**DOI:** 10.1007/s12185-022-03427-4

**Published:** 2022-08-09

**Authors:** Semra Aydin, Roberto Passera, Matilde Scaldaferri, Chiara Maria Dellacasa, Marco Poggiu, Francesco Cattel, Francesco Zallio, Lucia Brunello, Luisa Giaccone, Irene Dogliotti, Alessandro Busca

**Affiliations:** 1 Department of Oncology, Hematology, Immuno-Oncology and Rheumatology, University Hospital of Bonn, Venusberg-Campus 1, 53127 Bonn, Germany; 2 Department of Oncology, Hematology, A.O.U. Città Della Salute e Della Scienza, Turin, Italy; 3 Department of Medical Sciences, A.O.U. Città Della Salute e Della Scienza, University of Torino, Turin, Italy; 4 S.C. Clinical Pharmacology, A.O.U. Città Della Salute e Della Scienza, Turin, Italy; 5 Department of Oncology, SSD Stem Cell Transplant Center, A.O.U. Città Della Salute e Della Scienza, Turin, Italy; 6 Department of Hematology, SS Antonio & Biagio and C. Arrigo Hospital, Alessandria, Italy

**Keywords:** Sorafenib maintenance, FLT3–ITD mutation, Acute myeloid leukemia, Hematopoietic stem cell transplantation

## Abstract

In a retrospective analysis, 21 acute myeloid leukemia patients receiving single-agent sorafenib maintenance therapy in complete remission (CR) after hematopoietic stem cell transplantation (HSCT) were compared with a control group of 22 patients without maintenance. Sorafenib was initiated a median of 3 months (IQR: 2.3–3.5) after allogeneic HSCT with a median daily dosage of 400 mg (range: 200–800) orally, and lasted a median of 11.3 months (IQR: 3.3–24.4). No significant increase in graft versus host disease or toxicity was observed. Adverse events were reversible with dose adjustment or temporary discontinuation in 19/19 cases. With a median follow-up of 34.7 months (IQR: 16.9–79.5), sorafenib maintenance significantly improved cumulative incidence of relapse (*p* = 0.028) as well as overall survival (OS) (*p* = 0.016), especially in patients undergoing allogeneic HSCT in CR1 (*p* < 0.001). In conclusion, sorafenib maintenance after allogeneic HSCT is safe and may improve cumulative incidence of relapse and OS in FLT3–ITD-mutated AML.

**Supplementary Information:**

The online version contains supplementary material available at 10.1007/s12185-022-03427-4.

## Introduction

Allogeneic hematopoietic stem cell transplant (HSCT) has become the acknowledged standard for FMS-like tyrosine kinase internal tandem duplication (FLT3–ITD) harboring acute myeloid leukemia (AML) [1] based on two large studies, showing a long-term overall survival (OS) of > 50% for patients receiving HSCT in first complete remission (CR) [2, 3]. Since the RATIFY trial [4] reported a statistically significant overall survival benefit for midostaurin combinations in induction/consolidation chemotherapy and the ADMIRAL trial [5] showed a significant survival advantage for gilteritinib alone over salvage chemotherapy in relapsed/refractory FLT3-mutated AML patients, routine incorporation of FLT3 inhibitors in these patients is evolving. However, in the post-transplant setting, scarce clinical data are available about the interaction of FLT3 inhibition and allo-immunogeneity. Sorafenib is an oral multi-kinase inhibitor targeting not only FLT3 but also vascular endothelial growth factor (VEGF) receptors, c-Kit and platelet-derived growth factor (PDGF) receptors, all of which are expressed on AML and bone marrow stromal cells [6–9]. Scattered studies investigated the use of sorafenib as maintenance treatment after allogeneic HSCT. Preliminary results suggest prolonged OS and progression-free survival (PFS) [10–14]. Tyrosine kinase inhibition (TKI) seems to synergize with the graft versus leukemia effect (GVL) leading to a better leukemia control [15]*,* apparently without increase in graft versus host disease (GVHD) [16]*.* Of note, sorafenib toxicity seems to be manageable even in this group of fragile patients.

These preliminary data prompted us to compare retrospectively a cohort of 21 FLT3–ITD-mutated AML patients receiving sorafenib maintenance after allogeneic HSCT with a control group of 22 FLT3–ITD-mutated AML patients, who did not receive any maintenance treatment. The question was whether sorafenib maintenance influenced OS by graft versus leukemia synergy and if this synergy was related to an increased incidence of GVHD.

## Patients and methods

A total of 43 consecutive patients with FLT3–ITD-mutated AML were treated from 2014 to 2021 in two Italian hematology departments, namely the Stem Cell Transplant Center in Turin (*n* = 42) and the Azienda Ospedaliera SS Antonio e Biagio e C. Arrigo in Alessandria (*n* = 1) and were included in the present study. For homogeneity reasons, FLT3–tyrosine kinase domain (TKD)-mutated patients were excluded. At the time of data collection, allelic ratio quantification was not performed in both centers. Sorafenib maintenance treatment was administered to 21 patients after allogeneic HSCT (hence referred as sorafenib group), whereas 22 patients did not receive sorafenib maintenance (hence referred as control group). Since the use of sorafenib as post-transplant maintenance is off-label according to the indications authorized in Italy, each clinical case was reviewed and authorized by the Hospitals Commissions for Off Label Use of Drugs. Due to the retrospective observational nature of this study and according to Italian law (Italian Drugs Agency-AIFA, Guidelines for Observational Studies, 20 March 2008), no formal Institutional Ethic Committee/Institutional Review Board (IEC/IRB) approval was required. The study was performed in accordance with the principles stated in the Declaration of Helsinki. Before starting sorafenib, personal informed consent for off-label treatment was obtained from each patient. Hematologic CR defined according to current ELN criteria [17] and a full donor chimerism were required before treatment initiation. Patients in complete response with incomplete hematologic recovery (CRi), partial remission (PR), and morphologic leukemia-free state (MLFS) were included in the analysis. While CRi and MLFS patients were considered as CR patients, patients with PR were included in the primary refractory (PIF)/relapsed group. Sorafenib was administered orally as a single agent and adjusted to the best tolerated dose. The severity of adverse events was graded according to the National Cancer Institute Common Toxicity Criteria (CTCAE, version 4.0). GVHD was defined and classified according to standard criteria [18]. Neutrophil engraftment was defined as the first day of 3 consecutive days with an absolute neutrophil count > 500 × 10^6^/L, whereas platelet engraftment was defined as the first day of 7 consecutive days with a platelet count > 20.000 × 10^6^/L.

## Statistical analysis

The primary endpoint was OS, defined as the time from HSCT to death from any cause. OS was investigated either by the Kaplan–Meier method (comparing survival curves across groups by the log-rank test) or by the Cox proportional hazards model (comparing the two arms by the Wald test and calculating 95% confidence intervals). The OS curve stratified by sorafenib maintenance administration was estimated by a 3 months landmark point, due to the violation of proportional hazard assumption. The potential impact on OS was tested for following risk factors: age at HSCT (> 50 vs ≤ 50 years), recipient gender (male vs female), cytogenetic risk (high vs intermediate vs low), donor type (haploidentical vs matched sibling donor (MSD) vs MUD), disease status at HSCT (CR2/PIF/relapse vs CR1), conditioning regimen (myeloablative vs reduced intensity), acute (grade II/IV vs 0/I) and chronic GVHD (moderate/severe vs no/mild) occurrence, sorafenib maintenance administration (yes vs no), sorafenib administration before HSCT (yes vs no), duration of sorafenib administration (≥ 12 vs < 12 months), and start of sorafenib administration (≤ 2 vs ≥ 3 months from HSCT). The disease status at HSCT, the occurrence of acute/chronic GVHD, and the sorafenib maintenance administration were treated as time-dependent variables.

The secondary endpoint was the cumulative incidence of relapse (main event), using death without relapse (non-relapse mortality, NRM) as its competing event, while the cumulative incidence function was compared across the groups by the Gray test. Patients characteristics were reported using Fisher’s exact test for qualitative variables and the Mann–Whitney test for quantitative ones, described as median (inter quartile range-IQR). All reported *p* values were obtained by the two-sided exact method, at the conventional 5% significance level. Data were analyzed as of January 2022 by R 4.1.2. (R Foundation for Statistical Computing, Vienna-A, http://www.R-project.org).

## Results

### Patients’ characteristics

Baseline clinical and molecular characteristics of the patient cohort are summarized in Table 1. Of note, the number of intermediate- and high-risk patients as well as disease stage at HSCT was not significantly different in both groups: 19/21 (90.5%) patients in the sorafenib group and 18/22 (81.8%) patients in the control group were grafted in CR. Variables such as conditioning regimen, donor type, GVHD prophylaxis, number of infused CD34^+^ and CD3^+^ cells, as well as the median time of neutrophil and platelet engraftment were comparable in both groups. All patients received a T-cell-repleted graft, and in all patients, peripheral blood was the preferred stem cell source. A myeloablative conditioning regimen was used in 14/21 (66.7%) patients in the sorafenib group and in 18/22 (81.8%) patients in the control group. GVHD prophylaxis included methotrexate (MTX) and cyclosporine A (CSA) in matched sibling donor transplants, rabbit anti-thymocyte globulin and CSA-MTX in unrelated donor transplants, and tacrolimus-mycophenolate mofetil (MMF) combined with post-transplant cyclophosphamide in haploidentical transplants. Four patients received the combination of tacrolimus or CSA with MMF. Moreover, 10/21 patients in the sorafenib group and 14/22 patients in the control group had received FLT3 inhibitors before transplant.

**Table 1:** Baseline patients’ and transplant characteristics

Variable	All patients (*n* = 43)	Sorafenib (*n* = 21)	Control (*n* = 22)
Age at HSCT, years	55 (21–69)	55.3 (21–69)	55 (23–69)
Male	26 (60.5)	14 (66.7)	12 (54.5)
Cytogenetic risk, available	38 (88.4)	21 (100)	17 (77.3)
Favorable	1 (2.9)	1 (5.9)	0
Intermediate	32 (74.4)	18 (85.7)	14 (63.6)
Adverse	5 (11.6)	2 (9.5)	3 (13.6)
NPM1, available	43 (100)	21 (100)	22(100)
Mutated	27 (62.8)	14 (66.7)	13 (59.1)
Disease state at HSCT			
CR1/CR > 1	37 (86)	19 (90.5)	18 (81.8)
Refractory/relapsed	6 (14)	2 (9.5)	4 (18.2)
FLT3 inhibitors pre-HSCT	24 (55.8)	10 (47.6)	14 (63.6)
Conditioning			
MAC	32 (74.4)	14 (66.7)	18 (81.8)
RIC	11 (25.6)	7 (33.7)	4 (18.2)
Donor type			
MSD	12 (27.9)	5 (23.8)	7 (31.2)
MUD	23 (53.5)	11 (52.4)	12 (54.5)
Haplo	8 (18.6)	5 (23.8)	3 (13.6)
GVHD prophylaxis			
CSA-MTX-ATG	21 (48.8)	10 (47.6)	11 (50)
CSA-MTX	11 (25.6)	5 (23.8)	6 (27.3)
FK-MMF-EDX	7 (16.3)	4 (19)	3 (13.6)
CSA/FK-MMF	4 (9.3)	2 (9.5)	2 (9.1)
CD34^+^ cells infused, × 10^6^/kg	7.7 (2.8–20.1)	6,9 (3.8–10.5)	8.2 (2.8–20.1)
CD3^+^ cells infused, × 10^8^/kg	2.4 (1.3–6.7)	2.4 (1.3–4.4)	3.0 (1.6–6.7)
Time to neutrophil engraftment, days	16 (11–28)	16 (11–21)	17 (13–28)
Time to platelet engraftment, days	13 (8–29)	12 (8–35)	14 (9–29)
Outcome at last FU			
Dead	17 (39.5)	4 (19)	13 (59.1)
Alive	26 (60.5)	17 (81)	9 (40.1)

Data are *n* (%) or median (IQR, inter quartile range). *n* indicates number; *HSCT*, hematopoietic stem cell transplant; *NPM1*, Nucleophosmin 1; *CR*, complete remission; FLT3, FMS-like tyrosine kinase 3; MAC, myeloablative conditioning; RIC, reduced intensity conditioning; MSD, matched sibling donor; MUD, matched unrelated donor; *Haplo*, haploidentical; *GVHD*, graft versus host disease; *CSA*, Ciclosporin A; *MTX*, methotraxate; *ATG*, anti-thymocyte globulin; *FK*, tacrolimus; *MMF*, mycophenolate mofetil; *EDX*, Cyclophosphamid; *FU*, follow-up

### Treatment characteristics and toxicity of the patients receiving sorafenib

Table 2 summarizes treatment characteristics and toxicities occurring in the sorafenib group. Sorafenib was initiated after a median of 3 months (IQR: 2.3–3.5) after allogeneic HSCT with a median daily dosage of 400 mg (range: 200–800). The starting dose was at discretion of the referring physician. An adequate hematologic recovery and the absence of active GVHD treated with prednisone ≥ 0.5 mg/kg at time of sorafenib initiation was required. Sorafenib was administered for a median of 11.3 months (IQR: 3.3–24.4). A total of 14 patients experienced toxicities. As listed in Table 2, the skin and the gastro-intestinal (GI) tract were mostly involved. In some patients, even more than one organ was affected, for example one patient with skin toxicity developed concomitant grade 3 thrombocytopenia, another one displayed concomitant grade 3 liver function test elevation. Hence, adverse events led to dose reductions in 4 patients, temporary interruption in 5 patients lasting a median of 22 days (range: 13–30) and complete withdrawal of sorafenib in 4 patients. One patient with grade 2 toxicity developed abdominal pain and diarrhea: after sorafenib suspension, no improvement was observed. Of note, this patient developed signs suggestive for chronic GVHD (cGVHD) activity and required intensification of immunosuppressive therapy. Whether the GI symptoms were due to sorafenib toxicity or initial GVHD signs remained not fully elucidated. Among the other withdrawing patients, one refused to restart treatment after suspension for grade 2 GI toxicity whereas one patient discontinued the drug permanently due to disease progression and one due to skin-mouth-liver GVHD after DLI. Nevertheless, the majority of toxicities were documented as grade 1 (Table 2).

**Table 2:** Treatment characteristics of patients receiving sorafenib

Variable	*n* (%)
Time to sorafenib start, months	3 (2.3–3.5)
Grade I aGVHD at time of sorafenib start	5 (23.8)
Maximum dose, mg/day	
200	5 (23.8)
400	14 (66.7)
600	1 (4.8)
800	1 (4.8)
Toxicities	
None	7 (33.3)
Skin	5 (23.8)
Gastro-intestinal	4 (19)
Hematological	1 (4.8)
Combined/other	4 (18)
Time of toxicity onset, days	
Skin	14 (9–39)
Gastro-intestinal	42 (14–121)
Hematological	81
Combined	50
Grading of toxicity (CTCAE, version 4.0)	
0	7 (33.3)
1	12 (47.6)
2	2 (9.5)
3	2 (9.5)
Dose modification	
Reduction	4 (19)
Temporary discontinuation	5 (23.8)
Withdrawal	4 (19)
Duration of sorafenib maintenance, months	11.3 (3.3–24.4)

Data are *n* (%) or median (IQR, inter quartile range). *n* indicates number; *aGVHD*, acute graft versus host disease; *CTCAE*, common terminology criteria for adverse events; *HSCT*, hematopoietic stem cell transplant

Two patients had grade 3 toxicity: one developed grade 3 thrombocytopenia and sorafenib was withdrawn, while the other developed grade 3 liver toxicity with increase of the liver function tests: sorafenib was discontinued for 4 days, leading to normalization of the liver enzymes, so that sorafenib treatment could be resumed. In all patients experiencing adverse events, clinical improvement was observed after dose adjustments or temporary discontinuations.

### GVHD

At time of sorafenib maintenance initiation, 5/21 (23.8%) patients displayed grade I acute GVHD (aGVHD) of their skin: one patient responded to steroid therapy, while another developed cGVHD. During sorafenib maintenance, 7/21 (33.3%) patients experienced new onset of grade I/II aGVHD, manageable with observation or low-dose steroid therapy. No grade III/IV aGVHD was observed. In the control group, 11/22 (50%) patients developed aGVHD, of whom 8 (36.4%) were classified as grade I/II and 3 (13.6%) as III/IV aGVHD.

Overall, 10/21 (47.6%) patients in the sorafenib group developed cGVHD involving skin (*n* = 4), mouth (*n* = 4), and lung (*n* = 2): cGVHD was graded as mild in 2 cases, moderate in 6 cases, and severe in 2 cases. The incidence of cGVHD in the control group was comparable with the sorafenib group with 8/22 (36.4%) patients, of whom 4 mild, 3 moderate, and 1 severe cGVHD. Of note, most patients with moderate and severe cGVHD received donor lymphocyte infusions (DLI). In the sorafenib group, 3 (14.2%) patients received DLI for mixed donor chimerism (*n* = 1) or re-appearance of FLT3 mutation (*n* = 2). Their donor chimerism did not show any remarkable improvement after DLI, whereas patients with the re-appearance of FLT3 mutation achieved a molecular CR after DLI.

### Overall response rate

**Fig. 1:**
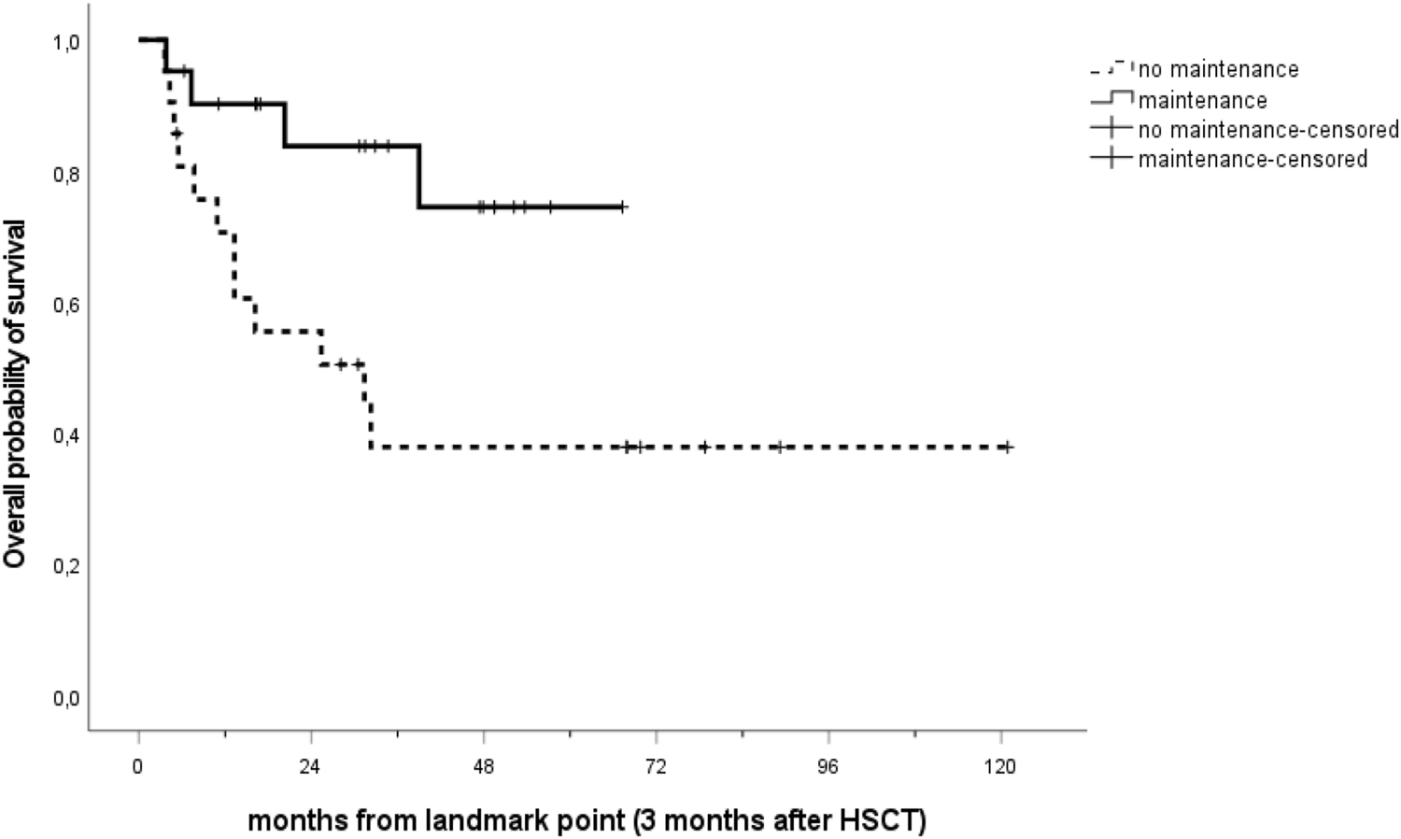
Overall survival of the sorafenib compared to the control group. Curves were stratified by sorafenib maintenance administration, and a landmark point at 3 months was applied. The median OS was 31.4 months in the control group, while it was not reached in the sorafenib group, *p* = 0.016. HSCT indicates hematopoietic stem cell transplant

The median follow-up of the entire cohort was 47.7 months (IQR: 28.1–67.3). Median OS was 31.4 months in the control group, while it was not reached in the sorafenib group, *p* = 0.016 (Fig. 1). Median follow-up of the control group was longer with 67.9 months (IQR: 30.5–78.8) compared to the more recent sorafenib group with 34.7 months (IQR: 16.9–49.5). At the time of last follow-up, 40.9% (*n* = 9) of patients of the control group and 81% (*n* = 17) patients of the sorafenib group were alive and still in complete remission.

**Fig. 2:**
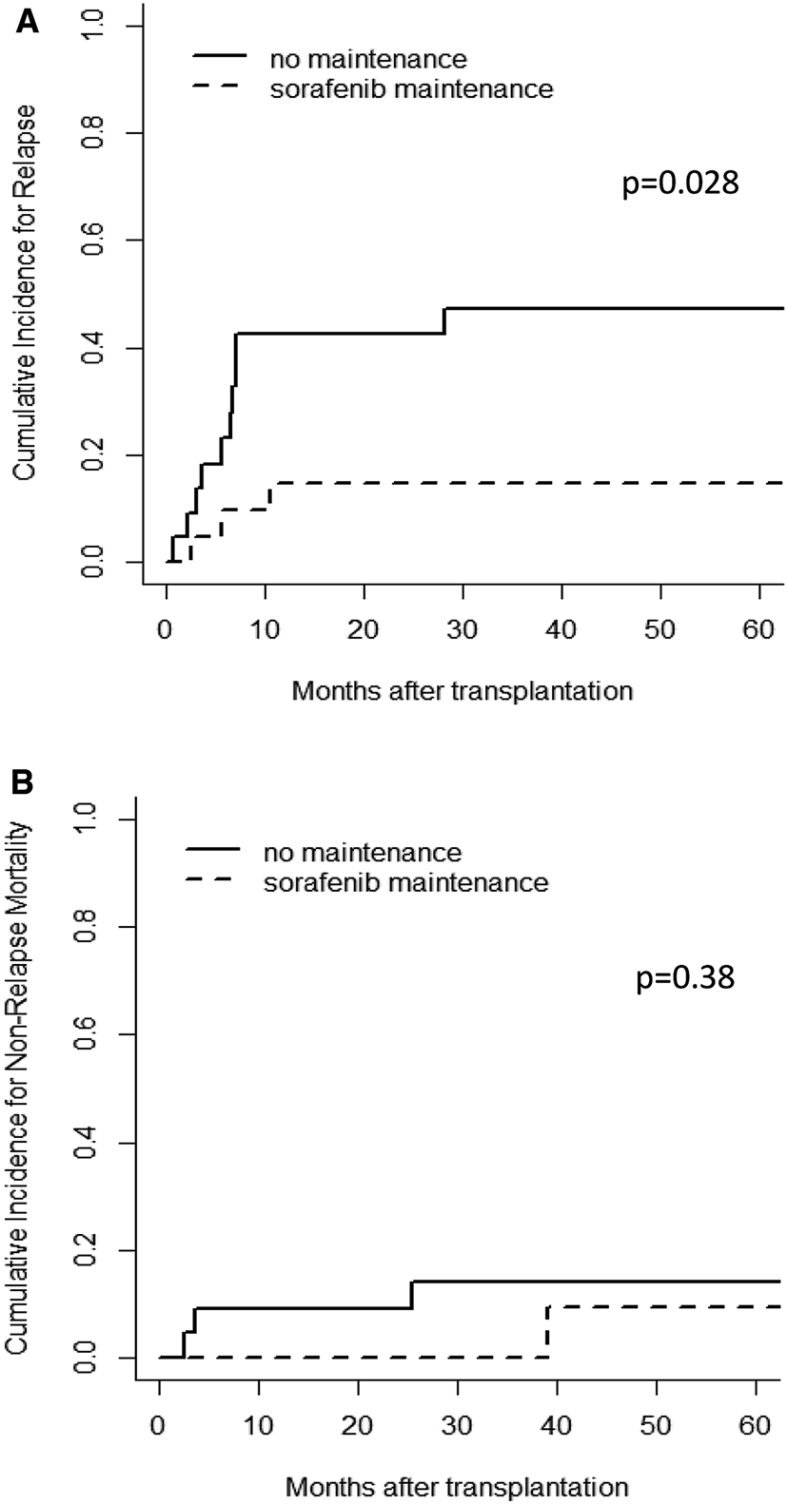
Cumulative incidence for relapse and non-relapse mortality stratified by sorafenib maintenance. A landmark point at 3 months was applied. **A** The cumulative incidence for relapse marked significantly higher in the control group compared to the sorafenib group, *p* = 0.028. **B** The cumulative incidence for non-relapse mortality was not significantly different between the control group and the sorafenib group, *p* = 0.38

The main cause for death in the control group was disease relapse. Cumulative incidence of relapse in the control group at one (42.4% vs 14.6%), two (42.4% vs 14.6%) or three (47.3% vs 14.6%) years resulted significantly higher than in the sorafenib group (Fig. 2a, *p* = 0.028), whereas no event for non-relapse mortality occurred in the sorafenib group at 1 (0% vs 9.1%), 2 (0% *vs* 9.1%), and 3 years (0% *vs* 13.9%) compared to the control group (Fig. 2b, *p* = 0.38).

**Fig. 3:**
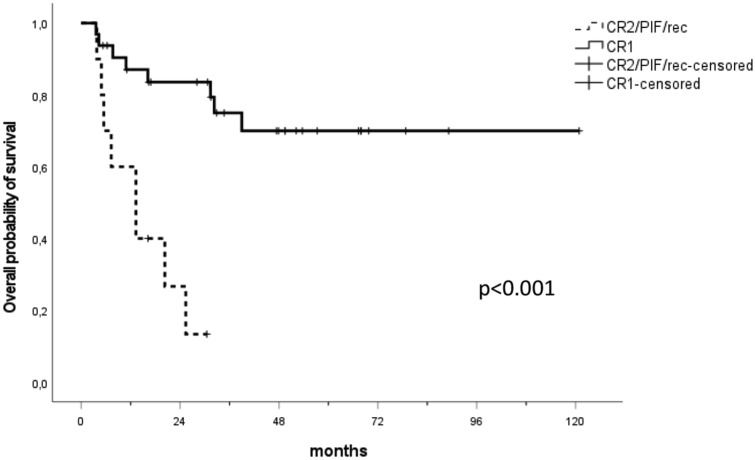
Overall survival of the CR1 patients compared to the CR2/PIF/relapsed group. A landmark point at 3 months was applied. The median OS of the CR1 patients (*n* = 32) was not reached and statistically significantly longer than the median OS of the CR2/PIF/relapse patients (*n* = 10) with 13 months, *p* < 0.001

Interestingly, when patients were analyzed according to their disease stage at HSCT, a survival benefit was significantly evident for patients in CR1. When updating the OS model and estimating the status at HSCT as a binary covariate at the same 3-month landmark point, the median OS for CR1 patients (*n* = 32) was not reached, while the median OS for CR2/PIF/relapsed patients (*n* = 10) was 13 months, *p* < 0.001 (Fig. 3). Of note, when stratifying the whole cohort by the occurrence of sorafenib maintenance, the difference in OS between CR1 *vs* CR2/PIF/relapse patients becomes highly significant, *p* < 0.001. In the sorafenib group (*n* = 21), the median OS difference was not reached for the CR1 patients versus 7 months for the CR2/PIF/relapse patients (supplementary figure A). Whereas, in the control group (*n* = 21), the difference was marginal, the median OS for CR1 patients was 32 months versus 13 months of CR2/PIF/relapse patients (*p* = 0.085, supplementary figure B).

## Discussion

FLT3 inhibitors are becoming crucial tools for curative treatment of patients with FLT3-positive AML. In the present study, we investigated the impact of oral sorafenib as single-agent maintenance therapy after HSCT in patients with FLT3–ITD-mutated AML. With a median follow-up of nearly 4 years, our results provide evidence that sorafenib, administered for a median of 11.3 months, significantly improves OS and reduces the cumulative incidence of relapse after allogeneic HSCT. The survival benefit seems to be even more significant in patients who undergo transplant in first CR.

Initially, Chen and colleagues reported in a pivotal phase I trial an impressive 1-year OS of 95% and a PFS rate of 85% with sorafenib maintenance in 22 FLT3–ITD-positive AML patients [10]. Subsequently, an interim analysis of the multi-center phase II-SORMAIN trial showed a 2-year leukemia-free survival of 85% for sorafenib maintenance of *n* = 43 patients versus 53.3% of *n* = 40 patients not receiving maintenance [19]. Regrettably, due to inadequate slow patient recruitment, the latter study was prematurely terminated. The results of the present study are in line with the SORMAIN trial: at time of last follow-up 81% of the sorafenib patients were still alive and in CR. Interestingly, in the SORMAIN trial 4 out of the 10 relapses in the sorafenib group occurred after the end of maintenance treatment, raising the issue of whether sorafenib maintenance should be continued longer than 24 months [19]. In contrast to the SORMAIN trial, in our study all 3 relapses occurred during sorafenib maintenance. Notably, all 3 patients had received HSCT not in CR, while all patients who received grafts in CR were able to maintain their remission with sorafenib maintenance, including one patient who died due to pneumonia.

An anti-leukemic synergism between sorafenib and allo-immune effects is supposed to be exerted by the stem cell graft [20–23]. In fact, myeloid leukemia mouse model studies showed that sorafenib increases IL-15 production in FLT-ITD-positive leukemic cells which synergizes with the allogeneic CD8^+^ T-cell response [22]. Enhanced IL-15 transcription levels led to enhanced mitochondrial spare respiratory and glycolytic capacity of CD8^+^ T cells, both important junction points in allogeneic immunity in human FLT3–ITD-positive AML cells [24]. Given the significantly higher advantage of sorafenib maintenance in CR1 patients in the present cohort, we presume that enforcement of GVL by sorafenib may be in part due to less treatment resistant leukemic cells, that are present immediately after induction chemotherapy. Strategies by resistant leukemic cells impairing the sorafenib mediated GVL effect are reported in a genetic deficient IL-15 receptor or in the production of lactic acid leading to T-cell dysfunction [25]. Moreover, less treatment toxicity before CR achievement may implicate less marrow niche damage in CR1 patients compared to CR2/PIF/relapse patients.

If sorafenib is able to potentiate the graft versus leukemia effect, does it also have a potential role in fostering the development of GVHD? Results of in vitro and preclinical murine experiments by Yokoyama and colleagues supported this hypothesis showing a significant increase of skin and liver GVHD, probably mediated by an increase in peripheral blood CD3^+^ T cells [26]. The exact mechanisms remain under investigation, but an increase of CD3-mediated T-cell proliferation by sorafenib seems to play an important role. A recent Chinese phase 3 trial [27] did not support this hypothesis, showing a consistently low incidence of acute (23% vs 21%) and chronic (18% vs 17%) GVHD in the sorafenib and their control group. This may be due to different GVHD prophylaxis protocols in the Chinese cohort. In the present study, the incidence of cGVHD in both groups was comparable (47.6% vs 36.4%), similar to the incidences reported in the SORMAIN trial [17] (62% in the sorafenib group vs 46.3% in the control group). Adverse events related to sorafenib administration were observed in 66.7% of our patients and were manageable by dose reductions and temporary discontinuation of the drug. Overall, four patients (19%) required complete withdrawal of sorafenib, similar to the rate reported in the SORMAIN trial (22%) [17] and by Chen et al. (22.7%) [10]. The rate of TKI discontinuation was significantly higher in the RADIUS trial [18], where 54.5% of the patients discontinued midostaurin maintenance due to toxicity, thereby suggesting that sorafenib has a better safety profile than midostaurin in the post-transplant setting.

Survival benefits by tyrosine kinase inhibition reported in literature have been described irrespective of the allelic burden. AML with low FLT3 allelic ratios (< 0.5) is currently classified by the European LeukemiaNet (ELN) as favorable risk; however, the majority of the data showing an adverse risk associated with high FLT3–ITD allelic ratio involved patients who did not receive FLT3 TKIs as part of the treatment [28, 29]. Therefore, it remains controversial if and how the FLT3–ITD allelic ratio at diagnosis may influence therapeutic strategies. Unfortunately, in the present study, FLT3–ITD allelic ratio was not available.

Few data on the development of sorafenib resistance are available. It has been suggested that AML chemotherapy may lead to elevated serum levels of FLT3-ligand that may produce autocrine stimulation of FLT3–ITD and therefore presumably induce kinase drug resistance [30, 31]. In the present cohort, 2/3 relapsing patients in the sorafenib group had received sorafenib before HSCT, whereas in the control group, 3/7 patients relapsing had received sorafenib prior to transplant. Certainly, main limitations of the current study are represented by the small cohort size, the retrospective nature, and a possible center effect.

In conclusion, the results of the present study show that sorafenib maintenance after allogeneic HSCT seems to be safe and may improve the outcome of FLT3–ITD-mutated patients in particular for patients grafted in CR. The incidence of both, acute and chronic GVHD, was not increased by sorafenib maintenance treatment. Additional multi-center prospective studies are mandatory to assess further the relevance of FLT3 allelic ratio and the role of next-generation FLT3 inhibitors in this post allogeneic HSCT setting.

## Supplementary Information

Below is the link to the electronic supplementary material.

Supplementary file1 (DOCX 2631 kb)
