# Supplementary material for: Sorafenib maintenance after hematopoietic stem cell transplantation improves outcome of FLT3–ITD-mutated acute myeloid leukemia
Source: Int J Hematol. 2022 Aug 9;116(6):883–91. doi: 10.1007/s12185-022-03427-4 (PMC9668769; doi:10.1007/s12185-022-03427-4)
Supplement: Supplementary file 1 — Supplementary file1 (DOCX 2631 kb) [file 12185_2022_3427_MOESM1_ESM.docx]

1. Sorafenib group


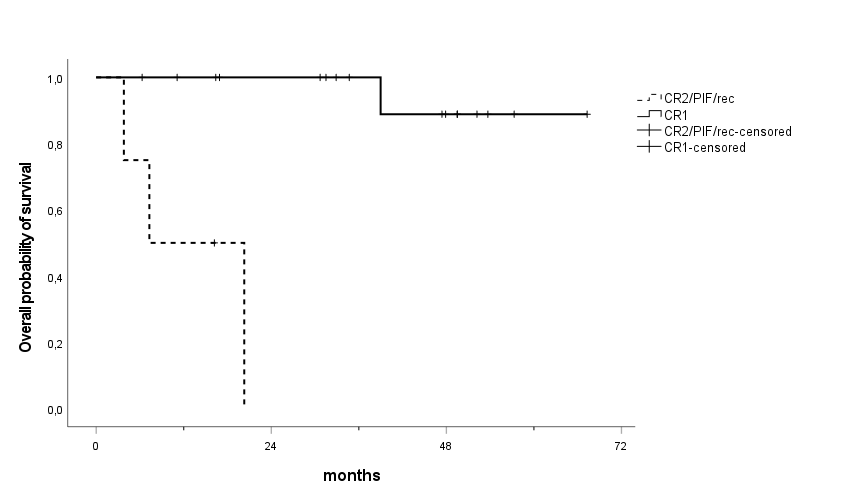

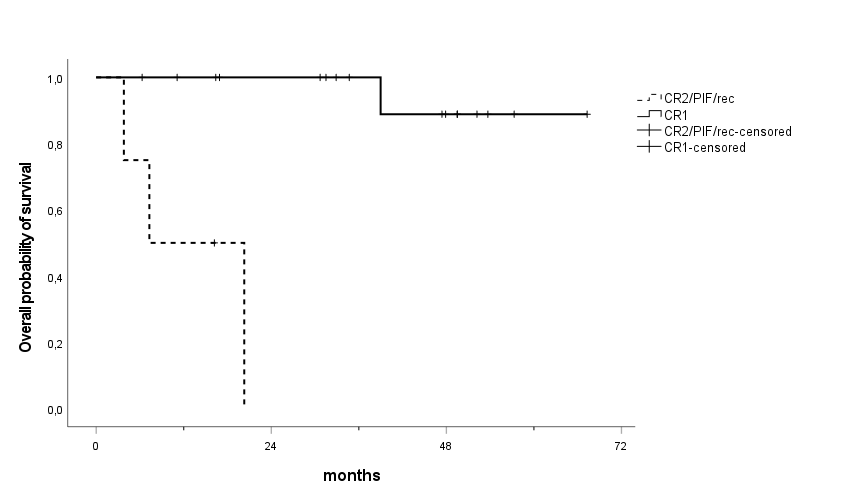


P<0.001

1. Control group


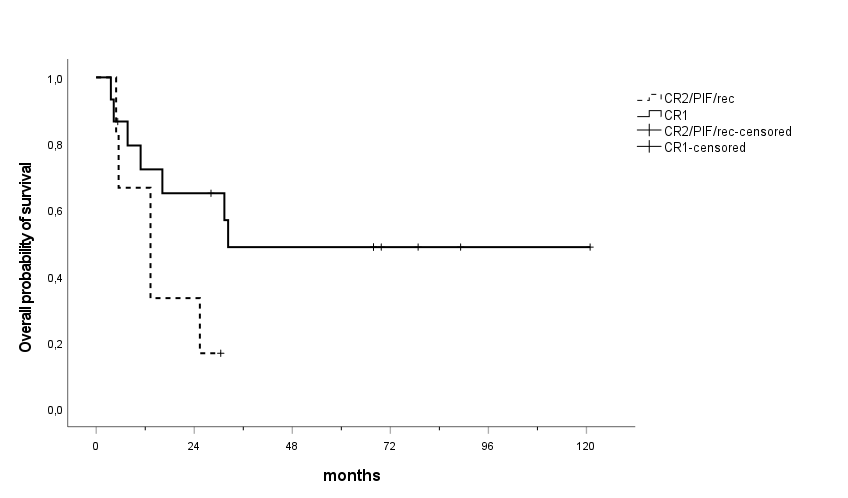

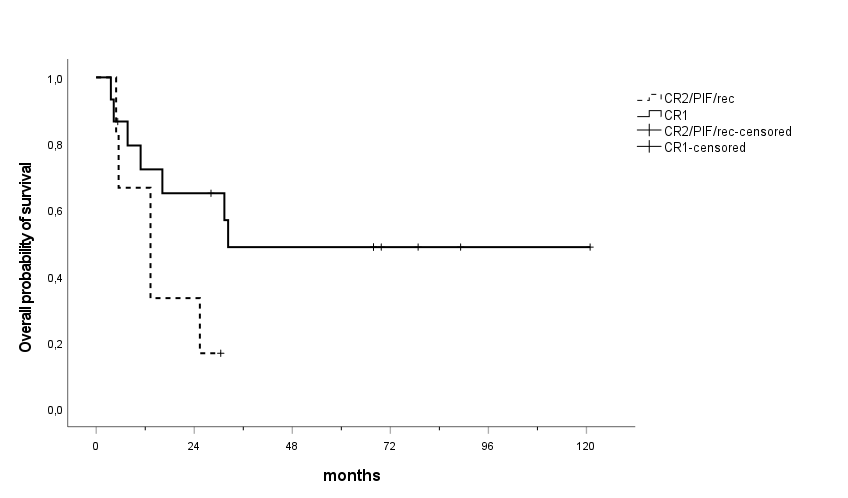


p=0.085

**Supplementary figure 1. Overall survival of patients’ subgroups stratified by disease stage at HSCT and sorafenib maintenance.** A landmark point at 3 months was applied. A) The difference between the CR1 *vs* CR2/PIF/relapse patients in the sorafenib group (n=21) with a not reached median OS for CR1 patients and 7 months median OS for CR2/PIF/relapse patients was highly statistically significant, p<0.001. B) The difference in OS between the CR1 *vs* CR2/PIF/relapse in the control group (n=21 pts) was not significant (p=0.085) with a median OS for CR1 patients of 32 months and the median OS for CR2/PIR/relapse patients of 13 months, respectively.
